# Continuous Intravenous Inotropes in Ward Units: Expanding Therapy Outside Intensive Care using a Safety-Oriented Protocol

**DOI:** 10.5935/abc.20190078

**Published:** 2019-05

**Authors:** Laura Caroline Tavares Hastenteufel, Nadine Clausell, Jeruza Lavanholi Neyeloff, Fernanda Bandeira Domingues, Larissa Gussatschenko Caballero, Eneida Rejane Rabelo da Silva, Lívia Adams Goldraich

**Affiliations:** Hospital de Clínicas de Porto Alegre, Porto Alegre, RS - Brazil

**Keywords:** Cardiotonic Agents, Dobutamine, Heart Failure/physiopathology, Inotropes, Milrinone

## Abstract

Selected clinically stable patients with heart failure (HF) who require prolonged intravenous inotropic therapy may benefit from its continuity out of the intensive care unit (ICU). We aimed to report on the initial experience and safety of a structured protocol for inotropic therapy in non-intensive care units in 28 consecutive patients hospitalized with HF that were discharged from ICU. The utilization of low to moderate inotropic doses oriented by a safety-focused process of care may reconfigure their role as a transition therapy while awaiting definitive advanced therapies and enable early ICU discharge.

## Introduction

In advanced heart failure (HF), patients with low output syndrome may benefit from intravenous inotropes to provide symptomatic relief and hemodynamic support with different purposes - stabilization of the acute setting, bridge to more definitive surgical therapies for advanced disease and palliation. Among patients admitted with decompensated HF, around 12 to 14% receive inotropes.^1^ However, the safety of inotrope use remains a concerning issue.^2^

In the acute setting, continuous inotropic infusions are usually initiated in intensive care units (ICU), where doses may be titrated with careful monitoring of pro-arrhythmogenic and vasodilatory effects until the patient is stabilized. Some patients may require longer periods of inotropic support, and, depending on their clinical status, may benefit from the continuity of inotropic therapy in a less intensive care setting. Our aim is to report the initial experience of a structured protocol for intravenous inotropic therapy in non-intensive care units, focusing on safety processes and end-points.

## Methods

We retrospectively reviewed all consecutive patients hospitalized with HF that were discharged from ICU on an intravenous inotropic infusion in our tertiary, academic hospital from July, 2015 to December, 2017. The strategy to promote discharge to the ward on inotropic therapy was supported by an institutional protocol, which is summarized in the Table 1. Briefly, stabilized HF patients receiving a low to moderate dose of continuous intravenous inotrope (dobutamine or milrinone) for different indications in the ICU were considered for transition of care to a ward unit equipped with cardiac telemetry, except if inotrope was intended for palliation, in which case telemetry was not used. Adverse events - defined as readmission to ICU due to worsening HF, atrial arrhythmia, ventricular arrhythmia requiring inotropic dose reduction, and infection related to central intravenous access - that occurred while the patient was receiving inotropic infusion in the ward were recorded. In-hospital outcomes (death, heart transplant, left ventricular assist device - LVAD - implant or weaned off inotropes), 30-day hospital readmissions, readmission for transplant and all-cause mortality up to a censoring date on December 31^st^, 2017 were recorded.

**Table 1:** Standard operating procedures for administration of continuous inotrope infusion in ward units

Eligibility
Patients that are clinically stable for more than 24 hours on a stable dose of one continuous intravenous inotrope and able to be discharged from the ICU.
Central venous catheter (preferentially PICC).
**Safety procedures**
Discharge to a continuous cardiac telemetry ward (except if intended for palliation).
Medical prescription including both inotropic dose (mcg/kg/min) and rate of infusion (mL/min).
Maximal recommended doses for inotropes in the ward: dobutamine = 5 mcg/kg/min; milrinone = 0.5 mcg/kg/min.
Fixed or gradually reduced the dose of inotrope, as clinically appropriate.
No dose increments in the ward (patient preferentially transferred back to ICU for dose augmentation).
Rigorous electrolyte targets (potassium 4.0-4.5 mmol/L; magnesium ≥ 2.0 mmol/L) and bicarbonate monitoring.
Systematic nursing evaluation of the patient and the administered drug according to the ward routines.
Daily patient assessment by the medical team.
**Considerations**
Exclusive intravenous access line for inotrope infusion.

ICU: intensive care unit; PICC: peripherally inserted central catheter.

### Statistical analysis

Categorical variables are presented as absolute numbers and percentages, and quantitative variables as mean ± standard deviation or median and interquartile range, as appropriate. A Kaplan-Meier curve was plotted for survival free from heart transplant or LVAD implant during follow-up, and cumulative incidence curves were calculated for all-cause mortality and heart transplant or LVAD using competing risk analysis with the R Software, version 3.4.4 (R Project for Statistical Computing, Vienna, Austria).^3^

## Results

We reviewed 28 patients with HF that were discharged from the ICU to the ward on intravenous inotropes after the protocol was created. Table 2 describes both patient and clinical care data during the inotropic support period. Figure 1A depicts in-hospital outcomes of patients according to intention for inotropic support.

**Table 2:** Characteristics of study patients and data pertaining the inotropic support

Characteristic	n = 28
**Baseline Characteristics**	
Age, years	54 ± 16
Male sex	20 (71.5)
Ischemic etiology of HF	16 (57)
Left ventricular ejection fraction, %	23 ± 7.5
History of atrial fibrillation	13 (46)
Implantable cardioverter defibrillator	13 (46)
Chronic kidney disease (GFR < 30 mL/min/1.73 m^2^)	7 (25)
**Inotrope infusion**	
**Intravenous inotrope**	
Milrinone	24 (86)
Dobutamine	4 (14)
**Inotrope dose**	
Milrinone, mcg/Kg/min	0.25 (0.2 - 0.34)
Dobutamine, mcg/Kg/min	5.7 (4.37 - 6.55)
Total duration of inotropic therapy, days	23.5 (13.75 - 45.5)
Duration of inotropic therapy at ward, days	10.5 (6.75 - 25)
**Venous access for drug infusion**	
Central venous catheter	4 (14)
Peripherally inserted central catheter	22 (79)
Peripheral venous access	2 (7)
Systolic blood pressure, mmHg*	93 ± 14
Diastolic blood pressure, mmHg*	59 ± 10

Data expressed as number (percentage), mean ± standard deviation or median (interquartile range).

* Blood pressures values at the initiation of inotropic therapy. Data from one patient not available. HF: heart failure; GFR: glomerular filtration rate.

**Figure:**
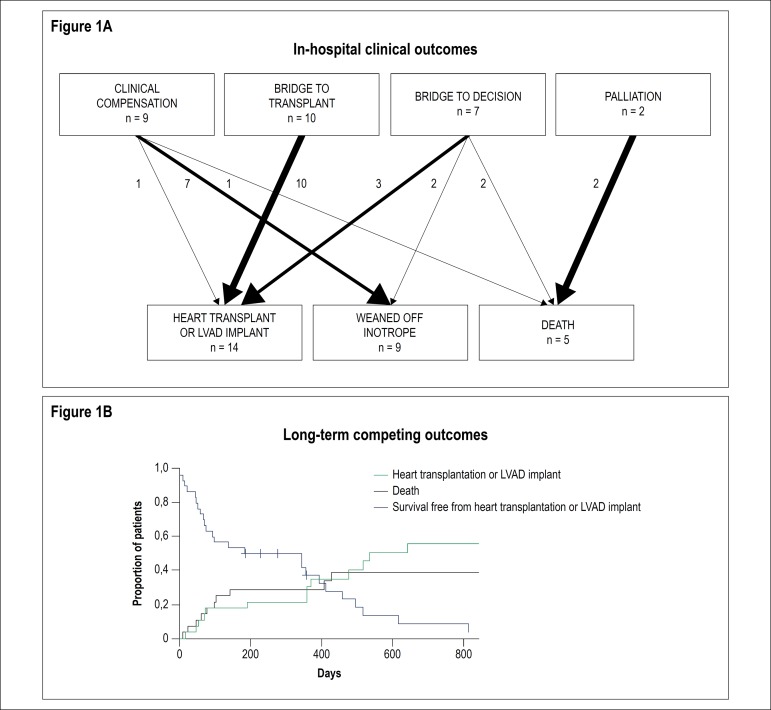
LVAD: left ventricular assist device.

The cohort was followed for a median of 154 days. Among those in whom inotropes were discontinued and that had hospital discharge free of heart transplant or LVAD implant (n = 8), two were readmitted for HF within 30 days. Competing outcomes for mortality during the follow-up period are demonstrated in Figure 1B.

During the period on inotropic support in the ward, nine patients returned to ICU due to worsening HF - two of those for worsening pre-existing atrial fibrillation or atrial flutter. No episodes of new atrial fibrillation or atrial flutter were observed. Six patients developed recurrent non-sustained ventricular arrhythmia, and inotropic dose was reduced; of those, four were hypokalemic (≤ 3.5 mmol/L) when arrhythmias were observed. One patient had a central venous catheter exit-site infection, and one had a peripherally inserted central catheter-related bloodstream infection. Seven events of protocol violation were identified: use of peripheral venous access for drug infusion (n = 2); and, increments in inotropic dose in the ward (n = 5). None of them incurred in clinical adverse events.

## Discussion

In the present report, we described our initial experience with a safety-focused protocol for the use of continuous intravenous inotropes in hospitalized patients with advanced HF outside the ICU. We demonstrated that a subset of clinically stable patients on inotropes may benefit from transition to a less intensive care setting following careful standard operating procedures, without a significant burden of adverse events. These safety measures are aligned with our institutional program for quality improvement.

Current guidelines indicate that inotropes can be used in specific clinical settings, especially cardiogenic shock or bridge therapy in patients with refractory HF awaiting heart transplant or LVAD. Also, those not candidates for definitive therapies could be considered for long-term inotrope as palliation.^4^ The use of intravenous inotropic agents remains controversial, as many reports have associated its utilization with unfavourable outcomes. A deleterious effect of its use on long-term mortality among patients discharged alive, however, has not been suggested by a recent European registry report.^1^

In this study, we describe a selected population of patients with advanced HF that has not been well-documented in most studies evaluating inotropes - mostly clinically stable hospitalized patients intended for inotropic wean or bridge to definite therapies. Concerning safety outcomes, most of the arrhythmogenic events occurred in the context of electrolyte disturbances, which can be potentially avoided with careful monitoring. Considering the growing HF severity and the inotrope potential as a bridge therapy in hospitalized patients, a contemporary approach to their utilization has been to focus on the safety profile of its use while maintaining the traditional goals of therapy (the 'until' therapy), as described by Stevenson.^5^ Avoidance of traditional high doses of inotropes, the administration under careful monitoring conditions and strict electrolyte correction strategies may allow broader use of these agents.

## Conclusions

A contemporary, safety-focused approach to the use of low to moderate doses of intravenous inotropic agents in less resource-intensive settings may be feasible, potentially reconfiguring the use of these agents in different scenarios, ranging from bridge therapy to end-of-life palliation.
